# Supporting Fair and Efficient Emergency Medical Services in a Large Heterogeneous Region

**DOI:** 10.1007/s41666-023-00154-1

**Published:** 2024-01-09

**Authors:** Francesca Da Ros, Luca Di Gaspero, Kevin Roitero, David La Barbera, Stefano Mizzaro, Vincenzo Della Mea, Francesca Valent, Laura Deroma

**Affiliations:** 1 Intelligent Optimization Laboratory, Universitá degli Studi di Udine, Udine, Italy; 2 DMIF, Universitá degli Studi di Udine, via delle Scienze 206, Udine, I-33100 Italy; 3 DPIA, Universitá degli Studi di Udine, via delle Scienze 206, Udine, I-33100 Italy; 4 Public Health and Hygiene, Azienda Ospedaliera Universitaria del Friuli Centrale, via Chiusaforte 2, Udine, I-33100 Italy

**Keywords:** EMS simulator, Multi-objective optimization, Real-world application, Decision support system, Fairness, Efficiency

## Abstract

Emergency Medical Services (EMS) are crucial in delivering timely and effective medical care to patients in need. However, the complex and dynamic nature of operations poses challenges for decision-making processes at strategic, tactical, and operational levels. This paper proposes an action-driven strategy for EMS management, employing a multi-objective optimizer and a simulator to evaluate potential outcomes of decisions. The approach combines historical data with dynamic simulations and multi-objective optimization techniques to inform decision-makers and improve the overall performance of the system. The research focuses on the Friuli Venezia Giulia region in north-eastern Italy. The region encompasses various landscapes and demographic situations that challenge fairness and equity in service access. Similar challenges are faced in other regions with comparable characteristics. The Decision Support System developed in this work accurately models the real-world system and provides valuable feedback and suggestions to EMS professionals, enabling them to make informed decisions and enhance the efficiency and fairness of the system.

## Introduction

Emergency Medical Services (EMS) are vital in providing timely and effective medical care and transportation to patients who require immediate medical attention, with the aim of reducing the risk of complications, disability, and death.

The complex and dynamic nature of EMS operations presents challenges for decision-making processes at different levels [7]. Indeed, from a strategic point of view, the main decisions are related to the design of the emergency network, i.e., the location of ambulance stations and the hospital emergency departments (EDs). Within tactical planning, the main concerns regard fleet sizing and management strategies (i.e., personnel rostering) and the allocation of ambulances to the stations. Eventually, at an operational level, issues such as ambulance relocation, ambulance dispatching (i.e., assigning the right ambulance at the right moment to an emergency call), and, more broadly, emergency management must be addressed.

Furthermore, decision-makers are required to balance conflicting objectives such as minimizing response times [25], maximizing patient survival rates, optimizing the use of resources, and ensuring fair and equitable access to EMS [2, 9], especially in the case of wide sparse areas with different demographic conditions.

Data-driven approaches are commonly used for the design of such a complex system; they involve data analytics to inform decision-makers about how to design the system. This includes collecting and analyzing a variety of data, i.e., historical response times, patient outcomes, resource utilization, and demographic and epidemiological information about the population being served.

The approach taken in this work shifts the focus towards an *action-driven* strategy, which involves an optimizer for automatically suggesting decisions and a simulator to determine their potential outcomes, in lieu of depending solely on static historical data. In other words, the aim is to dynamically determine the consequences of decisions and actions (e.g., the suitability of operations), rather than relying on static data and past patterns or trends (e.g., are there zones where delays in the rescues are frequent?).

While historical data is still utilized to design and validate the decision support tools, new ambulance location configurations are obtained through the optimizer, and the evaluation of *what-if* scenarios is conducted through simulation. Moreover, the simulator can be used also for supporting decisions at the tactical level, e.g., increasing or decreasing the fleet size or its temporal availability, and at the operational level, e.g., evaluating different dispatching policies.

The action-driven strategy aligns with the objectives of the EasyNet network project, which is a collaborative effort among seven regions in Italy, partially supported by the Italian Ministry of Health, meant to evaluate the effectiveness of Audit and Feedback interventions in reducing practice variations and improving quality and health outcomes. Audit and Feedback may be useful in improving the quality of healthcare, as it involves evaluating the performance of individuals or teams against established standards, previous measurements, or other professionals, and then providing feedback to help them improve [36]. Specifically, the project unit of Friuli Venezia Giulia (FVG) Autonomous Region is responsible for a work package that focuses on enhancing quality in emergency medicine.

This work presents a Decision Support System (DSS) specifically designed for this project, which enables the analysis of the current EMS management situation, the evaluation of alternative scenarios, and provides suggestions for possible decisions. By measuring the impact of these decisions, the system can provide valuable feedback to EMS professionals to improve the quality of the overall system and facilitate decision-making across all levels (strategic, tactical, operational).

As a case study, we focus on the aforementioned FVG region, which comprises a complex landscape consisting of urban, rural, coastal, and mountain areas, with different demographic characteristics and we base our tool on the analysis of emergency data collected in the years ranging from 2018 to 2020.

In particular, the DSS comprises a sophisticated simulator of EMS operations, which takes into account factors such as elaborate dispatching rules and optional low-priority service pre-emption, to improve response times and overall system efficiency. Moreover, the system has been designed to tackle the challenges arising from the wide and heterogeneous nature of the areas served by EMS, as well as the diverse resources available, such as ambulance types and personnel shifts. The use of Pareto multi-objective optimization allows for prioritizing both efficiency and fairness in the decision-support process, keeping them separate (rather than aggregated or expressed in a hierarchical form) so that the decision-maker can make a responsible and informed choice.

The system is built considering the details of real-world processes and is validated on real-world data. It features user-friendly dashboards bridging the gap between research and practice so that the EMS management is provided with actionable feedback on their decisions.

Our research offers the following main contributions:

(i) The decision support system is designed as an integrated suite of software tools, comprising an analysis module, an EMS simulator functioning as a *digital twin* of the system, and a multi-objective optimizer that supports automated decision making. This system is implemented within an Audit and Feedback project, aimed at identifying bottlenecks in the EMS and facilitating fair and efficient decision-making processes driven by actionable insights.
(ii) Unlike previous studies, our research focuses on a vast and heterogeneous region that encompasses urban, rural, coastal, and mountain areas, with particular emphasis on the challenges faced in accessing services in the mountains.
(iii) Our study offers a comprehensive examination of a particular case study, serving as a reference for the development of the decision support solution. Moreover, we propose a collection of essential performance indicators, with a specific focus on incorporating fairness-related metrics.
(iv) In order to encourage future research, we have made the EMS simulator, the optimizer, and the emergency data used publicly accessible to the research community. The remaining modules will be available upon request.

The remainder of this paper is organized as follows.

Section 2 presents an overview of literature work related to optimization, simulation, and other issues in the EMS context. Section 3 describes the audit phase conducted through an analysis of historical, geographic, and demographic data, later used for the validation of the DSS. Section 4 reports a set of indicators that describe the EMS performance considering both efficiency and fairness. Section 5 discusses the structure of the DSS, detailing the dashboard interface, the simulator, and the optimizer. The DSS is validated through different experiments in Section 6. Section 7 concludes the work by providing a discussion on current limitations and possible areas of improvement.

## Related Work

In this section, we overview the literature related to EMS. Specifically, we discuss the optimal placement of emergency vehicle locations (see Section 2.1) and we list several methodologies that have been proposed to account for fairness (see Section 2.2). Eventually, we present studies that leveraged multi-objective algorithms (see Section 2.3) and simulation-based approaches (see Section 2.4).

### The Emergency Vehicles Location Problem

Several contributions that discussed the (near-)optimal placement of EMS employ the concept of *coverage*, which involves the optimal distribution of resources to maximize the number of served locations [32, 54]. In the EMS context, service is represented in terms of locations reachable within a specific time or distance, usually modeled as a graph. The earliest study in this area is the Maximal Covering Location Problem (MCLP) [12], which aims at maximizing the demand coverage within a given response time. Later, Schilling et al. [50] extended MCLP to consider different types of vehicles, while [29] included the possibility for multiple ambulances and vehicles to cover a single zone. Despite providing only sub-optimal coverage, these models have a deterministic approach and fail to account for uncertainty. For example, they overlook scenarios where ambulances are already engaged in an emergency when a new call arises and needs to be addressed. To overcome this limitation, probabilistic models that incorporate uncertainty have emerged. One notable model is the Maximum Expected Covering Location Problem (MEXCPL) [15], which introduces the concept of the “busy fraction” to consider ambulance and vehicle availability in response to specific calls. By integrating uncertainty into the models, MEXCPL offers a more accurate representation of real-world emergency response systems. Additionally, MEXCPL provides a versatile framework applicable to other domains characterized by uncertainties, including disaster response and facility location.

Other authors optimize the deployment of vehicles by minimizing the response time. In this setting, the problem has been formalized as a “*p*-median problem”, which aims to locate *p* facilities to minimize the average distance between the demand points and their assigned facility [25]. Similarly, Ruslim and Ghani [48] considered isochrones^1^ analysis to determine a relocation policy, while [22] consider the Dynamic Location-Routing Problem (DLRP) to combine facility location and vehicle routing decisions and constraints to optimize the deployment of vehicles.

Most of the works deal with urban contexts, in which the underlying graph is quite dense because of the availability of an extended road network. The situation becomes a bit more challenging in the case of rural areas, where the locations are more sparse and the road network is not so extended. In this context [32] considered the location problem in a large-scale urban-rural area and proposed hybrid approaches, using both coverage and response time and considering an optimal trade-off between them.

Other complementary approaches aim to optimize the allocation of ambulances to different zones based on their expected workload, which can be measured in many ways: number of calls, expected travel time, or a combination of both. Zhu and McKnew [55], formalize the workload balancing problem by allocating ambulances to different zones to achieve balance with respect to the expected workload among different zones. Other approaches combine the expected workload, time, and coverage constraints [20, 21].

### Modeling Fairness

In EMS decision-making, optimal response time is crucial but it is also essential to be fair and equitable. Indeed, the *fairness* of ambulance location has become a prominent area of research. The objective is to distribute ambulance resources equitably, considering historical context, demand uncertainty, and operational constraints. This creates a fundamental challenge between efficiency and fairness, which has been ethically debated, specifically in the field of justice theories [46]. This issue can be framed as a conflict between utilitarianism, aiming to maximize the number of emergencies served within a given time, and egalitarianism, seeking equal access to medical care for all individuals. While an extensive analysis of these philosophical perspectives is not covered in this paper, it is important to acknowledge the underlying tension between these principles, which significantly impacts decision-making in practice [5, 24]. Studies have tried to reconcile efficiency and fairness in ambulance location models by adding objectives and constraints for equality. However, quantitatively defining fairness remains challenging due to the lack of consensus, resulting in various approaches with distinct perspectives.

To this end, it is crucial to consider two key points. Firstly, how to integrate a fairness metric into the model, which can vary depending on the selected approach. Secondly, it is equally important to determine which system performance indicators the fairness metric is describing.

Several measures have been proposed to capture fairness in facility location problems, including the range of the analyzed interval (e.g., the distance between the maximum and minimum response times), the deviation of the values from the center (e.g., mean absolute deviation), the deviation between each pair of values, and normalized measures (e.g., the Gini index).

Brotcorne et al. [9] and Akıncılar and Akıncılar [2] proposed robust ambulance location models that consider both fairness and uncertainty. These models aim to ensure that ambulance resources are distributed in a way that is both equitable and resilient to changes in demand.

Barbati and Piccolo [6] investigated fairness measures in the location problem and highlighted the use of the Gini index [23] as a promising approach. The Gini index, traditionally used to measure income inequality, can be applied to assess the relative imbalance among values in a distribution. In the context of the location problem, the Gini index serves as a measure of the fairness of the facility access across the population. A lower Gini index indicates a more equitable access, while a higher Gini index signifies greater inequality. By incorporating the Gini index into the decision-making process, decision-makers can effectively consider the consequences of their choices from an equity point of view.

Chanta et al. [11] proposed three bi-objective models that combine a utilitarian approach to maximize the expected number of calls responded by the system with fairness considerations. The models incorporate three different fairness measures: total number of uncovered demand in rural and urban zones, number of uncovered rural demand zones, and distance between uncovered demand zones and their nearest open stations. These fairness objectives are integrated into the optimization model using the $\epsilon$-constraint method. This method enables decision-makers to explicitly balance the utilitarian and fairness objectives, resulting in a set of optimal solutions that align with their preferences. By simultaneously considering both objectives, these models facilitate more informed and equitable decision-making.

Grot et al. [24] focuses on urban areas and proposes a model that maximizes the expected total coverage while incorporating two fairness criteria: the Gini coefficient and the Rawlsian criterion. The authors use the $\epsilon$ method to integrate these measures. The Rawlsian criterion evaluates the coverage of each demand sector by considering the worst-case scenario where a site is lost, thus accounting for the least advantaged. On the other hand, the Gini index measures the variation among different scenarios. These measures were chosen for their distinct emphasis on different aspects of fairness.

Finally, Jagtenberg and Mason [31] presents an example where the Bernoulli-Nash function is examined in the context of survival probabilities. This function can take two forms: the product of individual utilities and the geometric mean of all individuals. The product of individual utilities represents fairness as the probabilities of survival for each individual are multiplied together, prioritizing the most vulnerable members. On the other hand, the geometric mean calculates fairness by multiplying the survival probabilities and taking the reciprocal of the number of individuals. This method considers the overall survival probability of the entire group, making it more sensitive to changes in the survival probabilities of the entire group rather than focusing solely on the most vulnerable members.

### Multi-Objective Approaches

Since the aggregation of all the conflicting objectives into a single function prompts the decision-maker to establish their relative importance beforehand, an alternative to avoid premature commitments is to consider *multi-objective* methods. In this regard, numerous recent studies have leveraged this kind of approaches to address the EMS location problem.

Harewood [26] proposes a multi-objective version of the Maximum Availability Location Problem that maximizes population coverage within a given distance standard and level of reliability while minimizing the cost of covering the population. The method is tested using data from the Barbados Emergency Ambulance Service. Karatas and Yakıcı [33] presents a method to solve multi-objective facility location problems, focusing on public emergency service stations. The study integrates three well-known problems, namely the *p*-median problem, the maximal coverage location problem, and the *p*-center problem, to find a set of solutions for the three objectives altogether. The authors develop an algorithm that sequentially solves each individual objective problem using a combination of branch and bound and iterative programming techniques. Tsai et al. [51] proposes a method for ambulance allocation based on forecasting the distribution of EMS requesters. The approach uses a multi-objective ambulance allocation model solved by Particle Swarm Optimization. The method is tested using recorded historical data for EMS requesters in New Taipei City, Taiwan. Karatas and Yakıcı [34] presents a multi-objective facility location model for determining the number and locations of Temporary Emergency Service Centers for a natural gas distribution company in Turkey. The model considers three objectives: *p*-median, maximal coverage, and *p*-center to minimize transfer time. Olivos and Caceres [43] presents a multi-objective framework for solving an ambulance location problem in Antofagasta, Chile. The model considers mean response time, maximum response time, and demand not covered. The approach generates a set of solutions using an iterative $\epsilon$-constraint method. Historical data from 2015 and 2016 were used to generate demand and emergency locations. Wang et al. [52] proposes a multi-objective facility location problem to improve location reliability under facility uncertainty. The approach includes two objectives on reliability and coverage. A dual-population-based evolutionary algorithm is used to address the problem. The proposed method is applied to a real-world facility location with uncertainty of express cabinets in Tianjin, China.

### Simulation Models

Over the past decades, there has been a significant increase in the development of simulation models and simulation packages aimed at improving DSSs for fair and efficient EMS resource allocation [1]. These models are designed to provide a dynamical evaluation of the allocation of emergency medical resources such as ambulances by leveraging simulations, historical data, and providing an integration with optimization techniques.

The integration of simulation and optimization techniques is a crucial factor in the development of DSSs for various applications, including EMS resource allocation. Combining these two methodologies allows for a more comprehensive approach to resource management, providing valuable insights into the performance of different strategies and/or scenarios, and identifying areas for improvement [27].

Simulations play a crucial role in evaluating optimization techniques. In practical terms, these models function as digital twins of the system, facilitating a realistic evaluation of the decisions made although in a virtual environment, allowing decision-makers to quickly assess the effectiveness and efficiency of the available choices, possibly suggested by different optimization methods, across various scenarios. Through simulations, potential limitations or weaknesses in the solutions can be identified and addressed, leading to the development of resource allocation strategies that are both robust and reliable. Testing these strategies in simulated environments enables informed decision-making based on observed performance, resulting in improved resource management and system effectiveness. The utilization of historical data in simulations adds realism, allowing decision-makers to analyze patterns and trends over time, and providing valuable context for evaluating the optimization techniques.

Most EMS resource allocation simulation packages and frameworks primarily utilize Discrete Event Simulation (DES), which models system operation as a sequence of individual events occurring at defined time points. Each event triggers changes in the system’s internal state, and the simulation analyzes how the state evolves over time. DES is well-suited for complex systems like EMS resource allocation, where resources are dispatched and utilized in response to specific incidents or service requests. Although some frameworks adopt alternative simulation methodologies like agent-based simulations, DES remains the dominant choice in EMS due to its capacity to accurately represent the dynamic and stochastic nature of emergency medical service operations.

Numerous simulation packages and frameworks exist [1, 41, 53], some of them are open-source software whereas others are proprietary and developed for specific institutions, and not publicly available for general use.

BartSim [28] focuses on modeling and analyzing patient transportation systems, including ambulances and other medical transportation resources. Siren [27] optimizes the deployment and utilization of emergency medical resources, such as ambulances and paramedics, to enhance response times and system efficiency. Ambsim [38] is a DES framework that supports decision-making in ambulance service operations, providing insights into resource allocation and response time management. SIMEDIS [17] and its updated version, SIMEDIS 2.0 [16], are simulation tools aimed at modeling and optimizing emergency medical dispatching and resource allocation during mass casualty incidents. EMSSim [41] examines factors such as resource allocation, patient prioritization, and response times to improve the effectiveness of EMS operations. Lastly, JEMSS [47] is designed to support decision-making processes in emergency medical resource allocation and evaluate the effectiveness of different strategies in managing multi-agency EMS operations.

Among the optimization techniques that exploit simulation-based models, we recall Aringhieri et al. [3], who present a formulation for the ambulance location problem in an urban area in Italy, specifically in Milan. Their approach involves the use of integer linear programming models, whose results are further evaluated using an agent-based simulation model. McCormack and Coates [39] optimizes both the location of the stations and the vehicle fleet by employing a genetic algorithm and a simulation. Their objective is to maximize the expected survival probability across different patient classes. Bélanger et al. [8] introduces a recursive simulation-optimization framework, utilizing a DES in combination with a mathematical programming model to address location and dispatching decisions simultaneously. In a preliminary study, Da Ros et al. [14] determine the location of emergency vehicles by applying a multi-objective biased random-key genetic algorithm, which takes into account the results of simulations, such as the mean and the 90th percentile of response time, as well as a basic coverage measure.

## The Emergency Medical Service Context

The EMS faces many stochastic and interacting components, making designing robust decision-making tools a challenging task. Additionally, the influence of national regulations further compounds this complexity, making it difficult to separate the domain (i.e., EMS) from the case study (i.e., the case of an Italian region). However, even though this study focuses on Friuli Venezia Giulia, the concepts presented here can be extended to other regions with similar characteristics, especially considering those with a heterogeneous landscape.

This section presents the complex environment in which the DSS for EMS operates. The characteristics of FVG, hence its heterogeneous, diverse, and dynamic geography and demography are thoroughly analyzed (see Section 3.1). Resource limitations, such as the fixed number of available emergency vehicles per type (see Section 3.2), strict working hours, and a limited number of emergency care professionals (see Section 3.3), highlight the need to optimize the allocation of resources to ensure an efficient and fair service. Additionally, the hospital network’s configuration, organized into hubs (main centers) and spokes (satellite centers) presents further challenges to the EMS (see Section 3.4). Emergencies are inherently unpredictable and can vary significantly regarding urgency, location, timing, and other factors (see Section 3.5). The EMS is responsible for responding to emergency calls and providing care from the moment the call is received until the emergency vehicle returns to its original depot. However, response times can vary widely across different areas of the region, raising important questions regarding the trade-off between fairness and efficiency (see Section 3.6).

The current state of the EMS in the FVG region was established in 2015 through a data-driven approach. This involved dividing the region into zones and assigning a specific number of ambulances to each zone to ensure sufficient coverage for the population living in that area [44]. The regulation mandates that there should be one ambulance available for every 60,000 inhabitants or every 350 Km^2^. While some adjustments have been made to the basic model to consider scenarios involving multiple emergencies or other factors, the analysis has paid limited attention to the specific characteristics and requirements of individual zones and to the fairness aspects.

### Geography and Demography

The case study is about the Italian FVG region, composed of 215 municipalities with varying characteristics in terms of extension, environment, population, etc. Thus, in the EMS perspective, the region can be divided into four different areas, namely a limited number of cities (i.e., 5 municipalities with a population higher than 25,000 inhabitants), a vast rural area, an extensive and scarcely populated mountain area yet interested by tourism, and a set of touristic towns at the seaside (see Table 1 for the characteristics^2^ of the zones).

**Table 1:** FVG population and extension by area

Area	Municipalities	Population		Surface		Density
	#	#	%	km^2^	%	#/km^2^
Cities	5	413,647	35	242	3	1,709.3
Rural	125	613,074	51	3,233	41	189.6
Mountain	83	155,930	13	4,322	54	36.1
Seaside	2	14,644	1	135	2	108.5
Total	215	1,197,295	100	7,932	100	150.9

Data on the population is retrieved and surfaces are calculated from the specific dataset by [30]

The number of inhabitants and spatial extension alone does not provide a complete picture of the region since it attracts a significant number of tourists each year, with the seaside towns experiencing a peak of roughly 200,000 tourists daily [45].

**Fig. 1:**
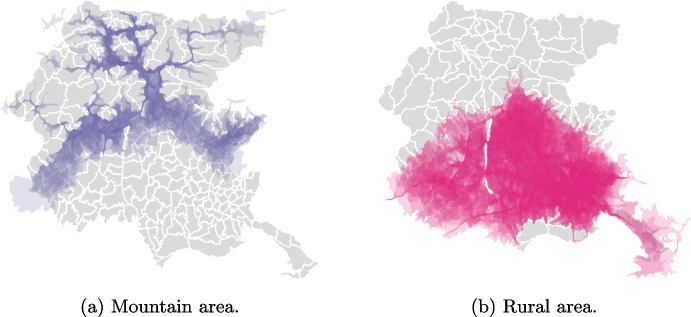
Isochrones at 18 minutes from a candidate ambulance location for each municipality

While rural and mountain areas share similarities in terms of their large size and sparse populations, there are significant differences in their road connections, which have important implications for EMS response times. As shown in Fig. 1, which illustrates the points reachable within 18 minutes^3^ from a candidate ambulance location for each municipality (i.e., isochrones), the limited and constrained road infrastructure in the mountain area results in longer travel times with respect to the rural areas. In this case, we intend candidate locations as possible locations for ambulance stations and they correspond to the position of the town hall of each municipality and the hospitals. As for the urban and coastal areas, the isochrones generally cover the entire surface within the standard response time.

### Vehicles

Medical vehicles can be of different types based on the service they offer, the way they are equipped, the team of professionals operating on them, and, overall, the use cases defined by national regulations. Namely, the Italian system comprehends Advanced Life Support (ALS) and Basic Life Support (BLS) ambulances, medical cars (MC), and medical helicopters (MH) (see Table 2). Medical cars are deployed in conjunction with ALS or BLS ambulances for joint rescue operations, ensuring the presence of a doctor or a highly specialized nurse during critical medical emergencies, particularly in cases of high-priority ones regarding specific time-critical pathologies such as strokes or heart attacks.

Vehicles can operate on different shifts. Generally speaking, they can work on a day shift (i.e., 8 am–8 pm) and a night shift (i.e., 8 pm–8 am). Furthermore, some vehicles are used in both the above-mentioned rosters (therefore, they are working on a 24-hour shift). Table 3 reports the vehicles operating in FVG on different rosters accounting for April 2022.

**Fig. 2:**
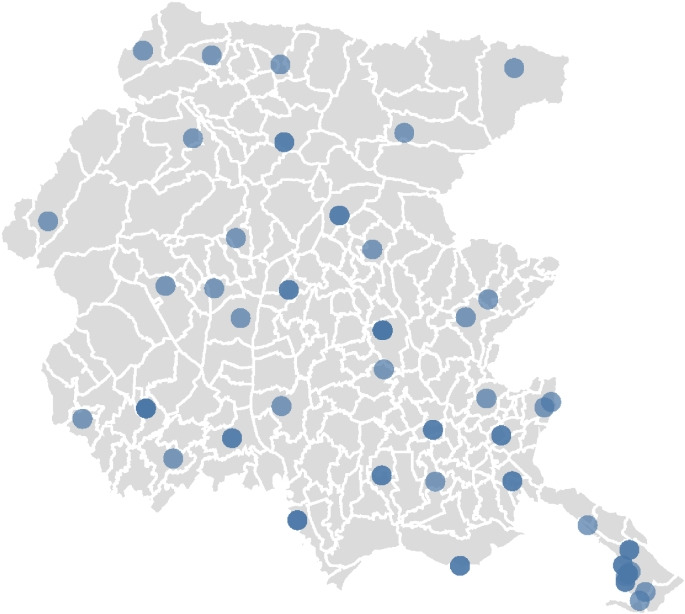
Current location of the ambulance stations in Friuli Venezia Giulia

The current location of ambulance stations is shown in Fig. 2. Besides the stations located at hospitals, each station hosts at most one ambulance.

### Personnel

The emergency care teams consist of a diverse group of professionals including emergency doctors, nurses, drivers, and volunteers who have received training in rescue operations and emergency medicine. These team members operate on various schedules or rosters. This arrangement is driven by the specific regulations governing the Italian EMS, which adhere to the “stay and play” system rather than the “scoop and run” one.

Under the “stay and play” system, the emphasis is placed on managing the emergency situation directly at the location where it occurs, rather than immediately transporting the patient to the nearest hospital, as is the case with the “scoop and run” system.

**Table 2:** Description of the emergency vehicles are per Italian and regional regulations [18]

Type	Equipment		Team (minimum requirement)	Patients’
	Basic	Advanced		transportation
ALS			Registered nurse, driver formed to handle rescue operations	
BLS			Volunteer, driver formed to handle rescue operations	
MC			Doctor or highly specialized nurse	
MH			Anesthesiologist and/or highly specialized nurse

**Table 3:** Emergency vehicles available in Friuli Venezia Giulia considering their shifts

Type	Day shift	Night shift	All-day shift
	(8 am–8 pm)	(8 pm–8 am)	
ALS	17	5	26
BLS	6	1	3
MC	3	1	3
MH	0	0	1

**Fig. 3:**
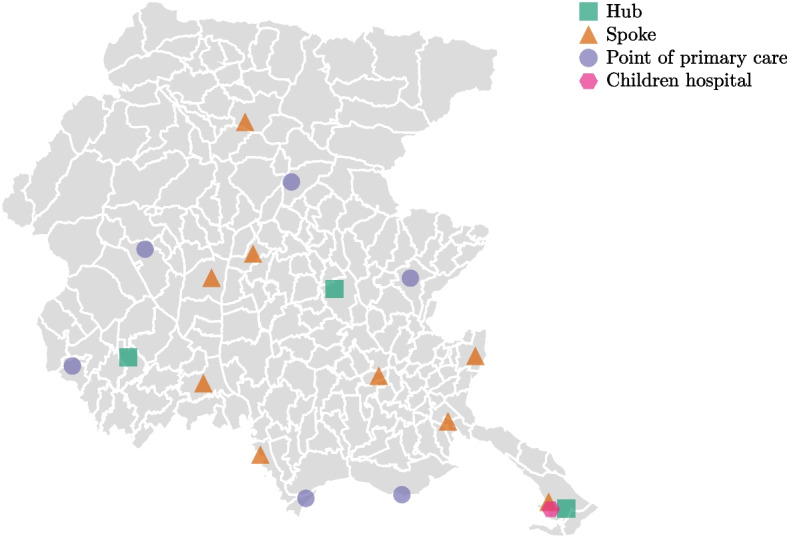
Location of healthcare facilities in Friuli Venezia Giulia, distinguishing among hospital types. The region accommodates four hubs, nine spokes, and six points of primary care. One of the four hubs is specialized in pediatric treatments

### Hospitals

Hospitals can be of different types based on the services they offer. Specifically, hospital distribution is related to a *hub-and-spoke network* organization [40]. Such a network comprises a main campus (i.e., a hub), which provides intensive and specialized medical services (e.g., heart surgery, neurosurgery), complemented by satellite sites (i.e., the spokes) [19], which supply only basic medical care. Additionally, a set of so-called *points of primary care*, hosting limited first aid care in rural and remote territories, is available (see Figure 3).

### Emergencies

Auditing the FVG EMS accounts also for understanding the spatiotemporal patterns of emergency occurrences. Thereof, we perform an analysis of emergency calls received between January 2018 and December 2020 and stored in the EMS database.

The emergency calls are categorized into four levels, namely red, yellow, green, and white, with red and yellow codes indicating the most urgent ones requiring prompt and advanced care. Each emergency call is also associated with a set of characteristics such as the location where it occurred, the number of people involved, the vehicle used in the rescue (e.g., ambulance, medical car, etc.), and the pathology^4^.

**Fig. 4:**
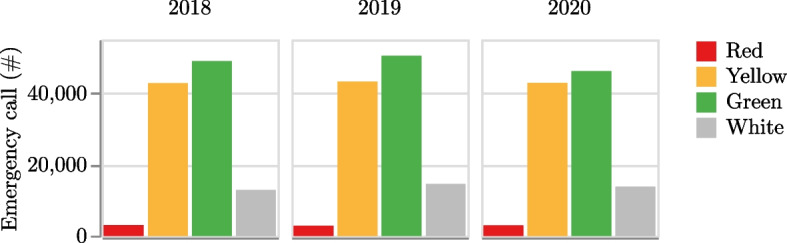
Number of emergency calls received in Friuli Venezia Giulia from January 2018 to December 2020 considering the urgency codes

Over the three-year period, a total of 323,968 emergency calls were received, with a nearly constant number of calls each year and a nearly constant distribution among urgency codes (see Fig. 4).

**Fig. 5:**
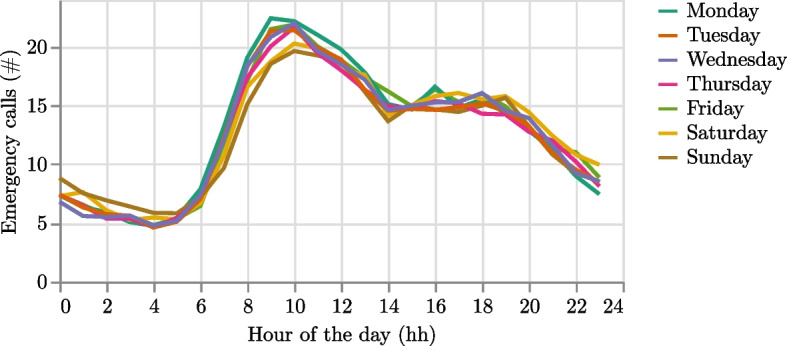
Mean number of emergency calls received in Friuli Venezia Giulia in the year 2019 analyzed by day of the week and hour of the day

Analysis of call volumes by time of day reveals that pressure on the EMS system is not evenly distributed over the 24-hour period, with higher call volumes in the middle morning and early evening (see Fig. 5).

**Fig. 6:**
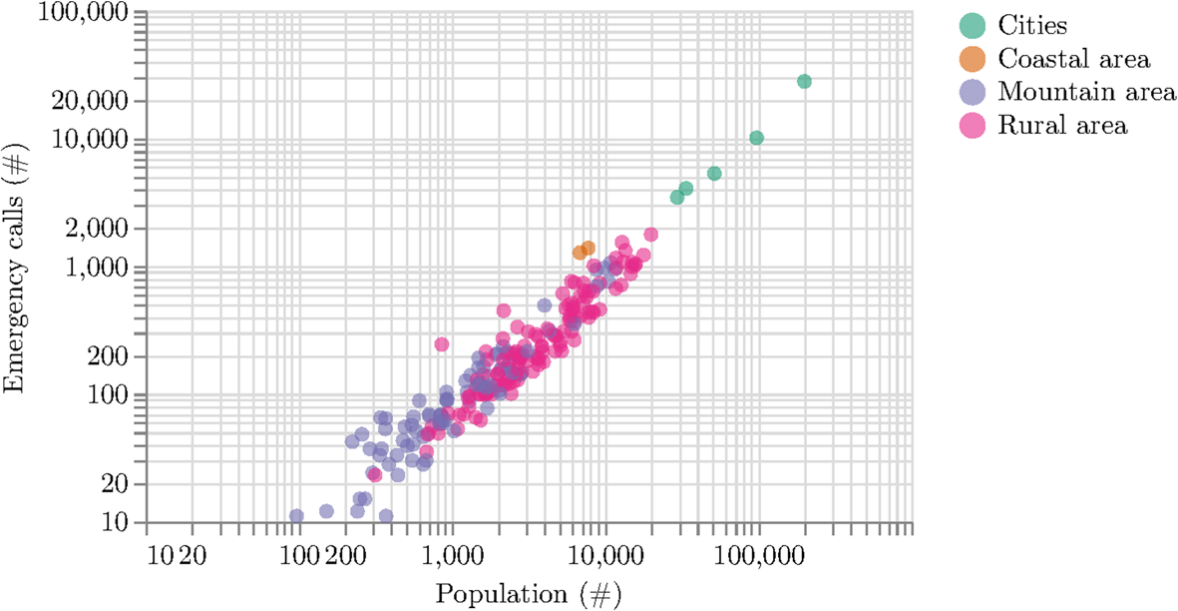
Relation between the number of emergency calls in Friuli Venezia Giulia and the number of inhabitants of the related municipality for the year 2019. The different areas are highlighted in different colors. The plot is in log-log scale

The spatial location of emergency calls is linked to population density, including both permanent residents and tourists. Figure 6 depicts the number of emergency calls against the number of inhabitants of the related municipality, with a clear relationship between the two in larger towns and cities. Instead, the relationship between emergency calls and population size is less clear in smaller villages.

**Fig. 7:**
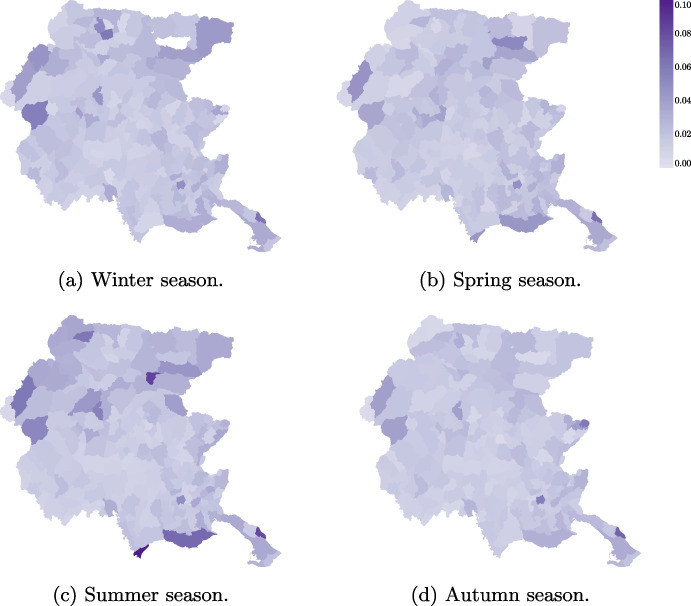
Number of emergency calls per capita received by each municipality during the year 2019 considering the four seasons

Analysis of emergency calls per capita by season shows higher call volumes in the mountain area during the winter months due to ski tourism, while summer months see increased call volumes in both the mountain and seaside tourist areas (see Fig. 7).

### Emergency Medical Service Processes

**Fig. 8:**
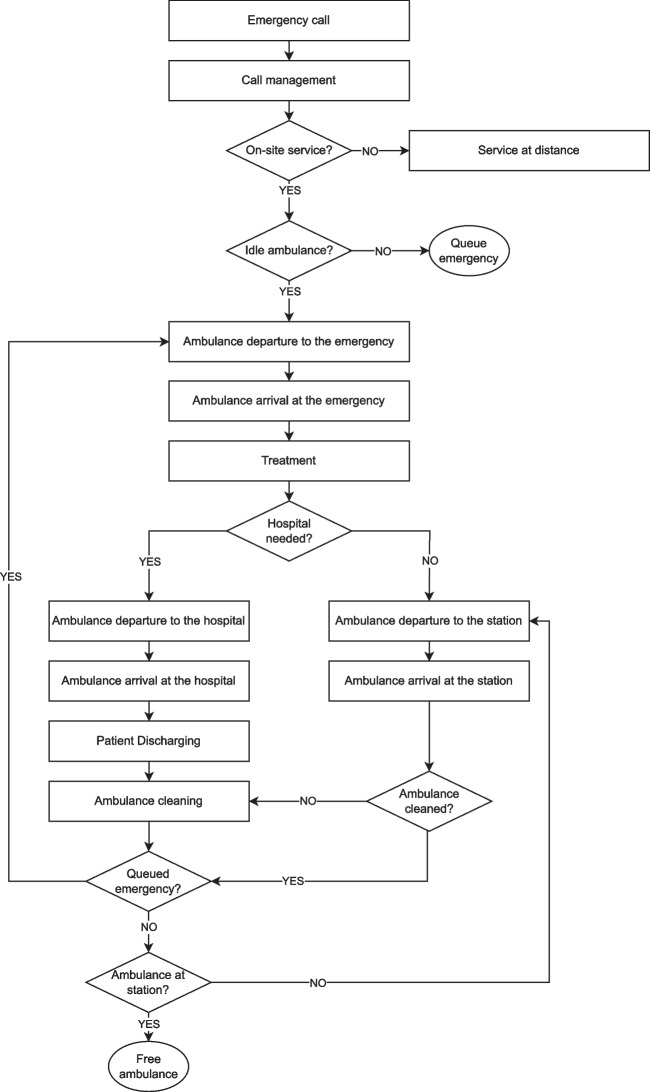
Emergency medical service decision process overview

The EMS plays a crucial role in responding to emergencies, from when a call is received to the return of the emergency vehicle to its station (see Fig. 8). According to the stay-and-play service model, the response time is a significant metric in describing the performance of the EMS processes. This is defined as the time elapsed between the occurrence of an emergency call and the moment when a vehicle reaches the emergency location (see Section 4.4). The response time is challenged in several ways, such as how distant the closer ambulance is, whether the closest ambulance is timely available or is serving another call, road connections and traffic status, weather conditions, etc.

**Fig. 9:**
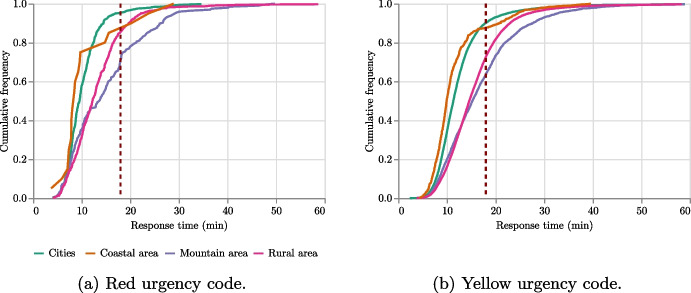
Comparison of the frequency of the response times (in minutes) among Friuli Venezia Giulia areas for the most urgent codes. The 18 minutes threshold is highlighted

Analyzing response times is particularly important for red and yellow urgency codes, as these indicate pathologies and events in which timely response (i.e., a response time within the 18 minutes threshold) is crucial for patient survival. Figure 9 reveals differences in these patterns among the four areas that compose FVG. While cities typically have the shortest response times, with most calls being served even within only 15 minutes for both red and yellow codes, the rural and mountain areas have the longest response times, thus failing to provide fair and efficient service to every patient.

Other possible time-related descriptors of the EMS include the time interval elapsed from the moment at which a call is received to the moment at which the first vehicle departs, the response time at the emergency location, etc. These indicators are not described in this paper, however, they have been considered in the design of the simulator.

## Emergency Medical Service Key Performance Indicators

When making decisions related to EMS, decision-makers must balance competing objectives, with efficiency and fairness being the most prominent considerations. Efficiency is achieved by ensuring that each person is served as quickly as possible, while fairness ensures that each person has equal access to the service.

The two above-mentioned perspectives are orthogonal. For instance, concentrating all ambulances in densely populated areas such as cities maximizes efficiency since sparsely populated regions would have lower service demands. However, this approach may result in longer response times for the people who are located far from the centralized areas. Conversely, achieving maximum fairness would entail having an emergency vehicle available in every municipality, but this is not feasible due to limited resources.

As discussed in Section 3, serving the mountain area poses significant challenges due to unique characteristics such as the road network, wide area, and low population density. Therefore, we have developed specific Key Performance Indicators (KPIs) to measure service access equality so not to discriminate the people living in that area.

In the following, we list a set of KPIs for assessing the level of service. Some of these objectives are accounted for in the optimization process (distinguishing day and night situation), while others are generated through simulation runs (see Sections 5.2 and 5.3 for details).

Specifically, we first introduce the KPI for ensuring fair coverage of the population (see Section 4.1), then some indicators for taking into account variations of the service levels (see Sections 4.2 and 4.3) and finally the efficiency measures (see Section 4.4). The formal definition of the KPIs is provided in Appendix A.

### Fair Coverage

We calculate the proportion of ambulances that can reach each resident within a specific municipality within the 18 minutes time threshold. This involves intersecting the isochrone centered at each ambulance location with the finer demographic data available (i.e., the census zones [30]) and dividing it by the total amount of population potentially covered by the ambulance. Disparities between municipalities are evaluated using the Gini index [23] (see Appendix A.1 for details).

Given our aim to ensure fair coverage, the objective is to minimize this index (i.e., a Gini index closer to zero indicates greater equality in the distribution of values among various municipalities).

### Population Coverage and Surface Coverage

We consider coverage in two ways, firstly in terms of population and secondly in terms of geographical zones. Namely, the population coverage considers how many inhabitants an ambulance serves, whereas the geographical coverage account for the surface that an ambulance can serve. The concept of service regards the number of inhabitants and the area that a given ambulance can reach within the aforementioned 18 minutes threshold (see Appendix A.2 for details).

The twofold point of view is due to the unpredictable nature of emergencies; in this way, we can account for calls received in zones that might be extensive but not highly populated (e.g., the mountain area). As BLS ambulances offer solely fundamental medical care (refer to Table 2), and are exclusively employed for critical emergencies when alternative vehicles are unavailable, we assess their coverage as a portion (70%) of the corresponding ALS service.

### Second Ambulance Distance

While the coverage measures introduced in the previous sections are effective in evaluating the quality of service in a given municipality, they assume that ambulances are always available, disregarding the possibility that an ambulance may be busy since it is responding to another emergency. In addition, the measures only consider a binary distinction between covered and not covered zones, without accounting for potential variations in service levels based on different response time thresholds. To overcome these limitations and explicitly take into account these circumstances, we consider the availability of the second closest ambulance for each zone in terms of road distance. This allows us to consider a scenario where the main ambulance is unavailable when a new call comes in.

The goal is to minimize the maximum of these values so that we can limit the effect on the worst possible scenario (see Appendix A.3 for details).

### Efficiency Measures

Finally, as one of the goals is to ensure the best possible level of service for each emergency, we consider the response times. Particularly, for each emergency code, we aim to minimize the elapsed time between the occurrence of an emergency call and the moment when a vehicle reaches the emergency location (see Appendix A.4 for details). Different urgency codes account for different levels of service (see Section 3.6 for details and Fig. 11b for an example).

## Decision Support System Architecture

**Fig. 10:**
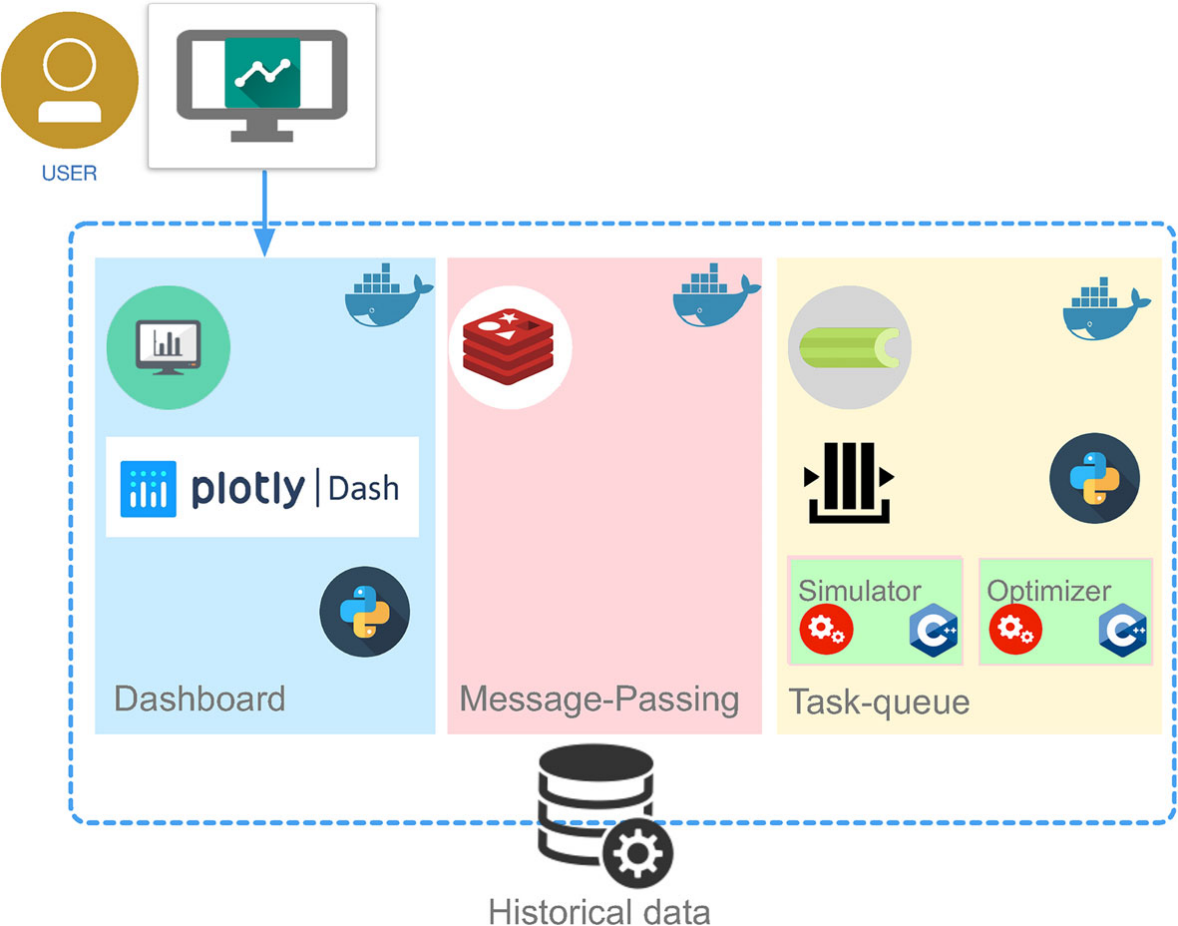
DSS Architecture

The DSS has been developed using a microservices architecture, with a focus on deploying it on a Docker platform to simplify the deployment process. The system’s structure is composed of three key microservices (see Fig. 10): a dashboard (see Section 5.1), a message-passing component, and a distributed task queue to manage long-running tasks, namely the EMS simulator (see Section 5.2) and the multi-objective optimizer (see Section 5.3).

The user interacts with the dashboard microservice, which is the only one exposed and is responsible for controlling the workflow and visualizing the result. The message-passing component is responsible for linking user actions on the dashboard to the third component, the distributed task queue, which runs the actual simulation and the decision-making optimizer.

The primary motivation behind selecting a containerized architecture based on microservices is to simplify the deployment process of the DSS software. This architecture enables convenient deployment, whether it is on-site or in a cloud environment. Additionally, it facilitates the seamless integration of diverse programming languages and technologies.

### Dashboard

The proposed framework establishes a connection between the decision maker, the simulator, and the optimization model through a dashboard, which serves as a human-computer interface. The dashboard main purpose is to provide a comprehensive view of the results of the analyses, while also allowing the user to control and display the results of the EMS simulator and optimizer. This way, the dashboard enables the decision-maker to build and evaluate alternative scenarios and make informed decisions through the visual representation of KPIs.

The dashboard has been implemented in Python, using the Plotly Dash interaction and visualization framework.

The interface reports the analyses for both the *as is* situation shown in Section 3 (i.e., the audit phase), and the outcomes of the *to be* scenarios through the execution of the EMS simulator. Moreover, the scenario can also be determined by the optimizer. The simulator and the optimizer are run asynchronously on a dedicated queue of potentially long-running processes.

**Fig. 11:**
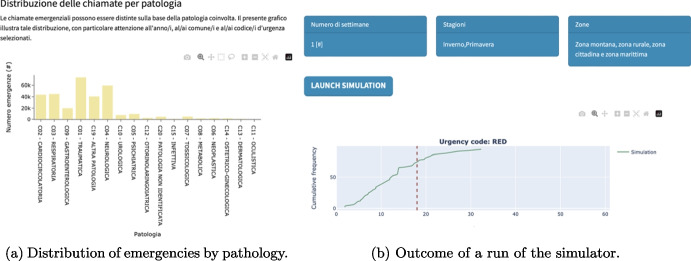
Examples of dashboard functionalities

The interface is localized in the Italian language; we show here a few examples of the core functionalities. Figure 11a reports an example of an analysis of the *as is* situation. The picture shows the distribution of emergency calls grouped by pathology. On the other hand, Fig. 11b reports an excerpt of the outcomes of a possible *to be* situation evaluated through a simulator run.

In a multi-objective optimization context, the dashboard also acts as a central hub that presents the Pareto front, enabling decision-makers to visualize and compare the trade-offs between conflicting objectives and identify the most desirable solutions (see Fig. 15 on page 22 for a visualization example).

Although the simulator and optimizer components are accessible to the decision-maker through the dashboard and the comprehensive microservices architecture discussed in Section 5, they can also be executed as individual offline modules (e.g., as for the validations performed in Sections 6.2 and 6.3).

Through the DSS dashboard, the decision-makers can edit the resource allocation scenarios, thus allowing the evaluation of choices at the strategic and tactical decision levels. For example, it is possible to relocate ambulances to different stations or extend the shift availability directly from the interface.

### Simulator

The EMS simulator is a Discrete Event Simulator and acts as a digital twin of the real-world system implementing the processes mapped and described in Section 3.6.

The simulator has been implemented in C++ using a customized version of the simcpp20 framework^5^, which is a coroutine-based discrete-event simulation framework for C++20. Initially, the system’s earlier prototype was developed in Python using the original SimPy framework, but due to unsatisfactory performances, we switched to C++.

The simulator receives two main inputs: a collection of geolocalized emergencies (e.g., in this study they are extracted from historical data), and a new allocation scenario comprising ambulance locations and personnel rosters. This way all the relevant decision points at the different decision levels are customizable. It then simulates the progression of the management of these emergencies, incorporating stochastic elements such as the time required to stabilize a patient before transportation to the hospital.

The output of the simulator is a log of the process for serving each emergency. This log is stored in a database and can be utilized to calculate time-based KPIs for the EMS system.

Our simulation package offers a distinct approach compared to those proposed in the literature, particularly in relation to JEMSS [47], which is the most similar to our framework. More in detail, our simulation package differs from existing approaches due to the following unique features: (i) we consider three types of emergency vehicles, incorporating the possibility of using a medical car in a joint service with traditional ambulance types; (ii) we account for different shift patterns for emergency vehicles, enabling users to customize vehicle availability based on specific customizable shifts (e.g., 00:00-24:00, 08:00-20:00, 7:30-19:30, etc.); (iii) we classify emergencies into four distinct urgency categories, providing a more granular view of the types of incidents requiring emergency medical services; and (iv) we categorize hospitals based on the services they offer, rather than solely on their geographical location. The inclusion of these features results in a simulation framework that is more flexible, comprehensive, and accurate. As a result, users can effectively address a wider variety of scenarios related to allocating emergency medical service resources.

In the following, we discuss the simulator by depicting its components (see Section 5.2.1), analyzing the fundamental procedures and decision protocols (see Section 5.2.2), and addressing the model assumptions (see Section 5.2.3).

#### Entities

The *emergency* entity in our simulator represents a medical event that requires attention. Each emergency has a set of static features that are received as inputs and remain constant throughout the simulation. These features include the arrival time of the emergency call, the location of the emergency (specified by longitude and latitude coordinates), the priority of the emergency (indicated by the color code according to the specifications of Section 3.5), and the type of hospital required for the specific medical condition (see Section 3.4). An emergency may or may not require transportation to a hospital.

During the simulation, other characteristics are collected, such as the time of various procedures related to the rescue and their durations (e.g., the time the service starts, the time of arrival at the emergency site, and the duration of the treatment at the location). The time for triage (i.e., the time taken by the operator to identify the situation’s urgency), treatment, patient drop-off, and ambulance cleaning are determined by random sampling a set of exponential distributions parametrized with values that are inferred from the analysis of historical data.

The state changes and multiple attributes of each emergency are logged during the simulation to ensure that the decision-maker has access to comprehensive information about the process for assessing the level of service.

The *ambulance* entity stands for a medical vehicle together with the linked resources. Each ambulance is associated with a type (see Section 3.2), and an operating shift (expressed as the time at which it starts working and the time at which it stops working). Furthermore, each ambulance is located at a specific station, identified by its longitude and latitude. During the simulation, as it happens for emergencies, a series of information is collected (e.g., the position at any time) and stored for analysis.

The *hospital* entity is described by its location and type (see Section 3.4) and is the target of the transportation according to the specific decision protocol.

The focus of the study is strictly on the service provided by emergency vehicles. Consequently, the simulator tracks the patient’s path up to the moment when the ambulance reaches the hospital, and the patient is taken care of by the Emergency Department.

#### Procedures and Decision Protocols

The *dispatcher* block connects the above-mentioned entities, by generating the emergency events and tracking ambulances availability. Broadly speaking, it acts as the operational emergency center that coordinates and manages the emergency situation of the region.

One of the crucial decisions that EMS must make is to determine the appropriate vehicle to serve a given emergency. This decision has a significant impact on the outcome of the rescue: timely and effective decision-making can determine whether a patient survives the emergency event or not.

The simulator employs a hierarchical approach for the dispatching decisions. Primarily, it takes into account the urgency of the emergency and matches it with suitable vehicle types. Secondly, it considers the distance of the available vehicles from the emergency location.

**Fig. 12:**
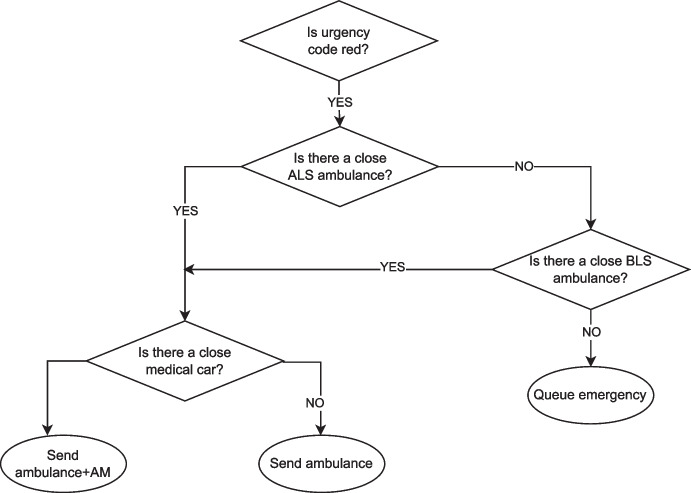
Decision process for the dispatching of emergency vehicles to serve an emergency call of urgency code red

Furthermore, an ambulance may consider one or more vehicles, as each ambulance may be coupled together with a medical car for the most urgent cases. For instance, Fig. 12 reports an example of the dispatching process for an emergency of red code.

Regarding the choice of the nearest ambulance, to speed up the computation, an initial filtering of potential vehicles is performed using the Haversine distance, considering ambulances within a given range from the emergency (the default is set to a 20km radius). In case of critical emergencies (i.e., red and yellow urgency code), if no vehicle is available within the range, the search is extended to the entire region. Subsequently, the dispatcher entity selects the vehicle or vehicles that are closest in terms of the actual road network from the initially filtered options.

Furthermore, the consideration of the availability of nearby emergency vehicles has led to the implementation of two distinct dispatching policies:

- *Policy without preemption*: A vehicle is deemed available only if it is waiting at a station.
- *Policy with preemption*: A vehicle is considered available if it is waiting, or it can be interrupted if currently engaged in a non-urgent service and is in close proximity, or has just completed a service at a hospital and is returning to its station.

The inclusion of the second policy introduces additional complexity to the dispatching process, thereby making also the simulation more intricate. However, this policy appears to align better with real-world practices, as outlined in the analysis reported in Section 6.2. This also highlights the possibility of evaluating operational decisions, which can be easily implemented in the simulator.

In situations where no ambulance is available to respond to an emergency call or when a more critical emergency arises and interrupts an ongoing service, the emergency is placed in a queue. As soon as a vehicle becomes available again, the queue is sorted based on the urgency, the waiting time, and the location of the emergencies. If a compatible emergency waiting in the queue is located nearby, it is selected to be served immediately. The selection process ensures that emergencies are chosen based on their priority, proximity to the available ambulance, and the amount of time they have been waiting in the queue.

In most cases, emergencies require transportation to a hospital for specific treatments. Depending on the type of treatment required (i.e., provided by a hub or a spoke hospital), the closest hospital of the suitable type is selected to provide the necessary care.

#### Limitations and Assumptions

The simulator effectively captures the complexity of the system and serves as a suitable digital-twin (see Section 6.2 for an evaluation of its accuracy). Nevertheless, it is important to note that it is based on a set of underlying assumptions, including the notion that each emergency is associated with precisely one code, and that follow-up calls are not received for all emergencies. Additionally, the simulator does not account for queues congestions at hospitals’ EDs and omits MHs, since the use of a helicopter is determined on a case-by-case basis by medical personnel for special circumstances, such as service to islands, mountain rescues, or situations where a timely response is of the utmost importance. Moreover, it is worth noticing that the helicopter is always used with other vehicles to provide support.

### Multi-objective Optimizer

In the DSS we adopt a multi-objective optimization perspective instead of a single-objective aggregated approach. This way, decision-makers can avoid making premature commitments regarding the trade-offs between conflicting objectives.

Specifically, the multi-objective optimizer focuses on facilitating an automated decision process designed to identify an optimal vehicle fleet location and determine personnel rosters. both day and night shifts. It offers the flexibility to explore different scenarios. For instance, it enables the optimization of existing vehicle utilization, uncovering potential areas for enhancement. Additionally, it allows for the examination of different combinations of vehicle numbers, shifts, or types, facilitating *what-if* analysis at a mixed level, incorporating strategic and tactical aspects. For example, it provides suggestions on where to relocate the vehicles if their number and temporal availability are increasing or decreasing.

The following information should be provided as input to the optimizer:

- *Candidate locations*: set of possible locations for the vehicles. Each location is associated with a set of characteristics, i.e., isochrones at different time intervals that are computed offline.
- *Vehicles information*: vehicles that need to be allocated. One must indicate how many vehicles of a given type can work in a given shift. It is assumed that the personnel assigned to each vehicle is available during that shift.
- *Region description*: the region we want to serve is divided into macro-portions, i.e., municipalities. Each municipality is composed of a set of zones, further characterized by a surface, a population, and therefore a density.

Similarly to the simulator, the optimizer is implemented using C++ for performance reasons.

The optimizer relies on *Local Search*, which is a metaheuristic optimization method that explores a solution space by iteratively making incremental improvements to an initial solution. The search starts from an initial solution, and it iteratively searches for neighboring solutions by applying local modifications. The search is guided by a cost function and proceeds until a satisfactory solution is found or a termination criterion is met.

In order to instantiate Local Search for the optimization problem at hand we describe the search space and the initial solution 5.3.1), then we recall the structure of the cost function (see Section 5.3.2), and we discuss the neighborhood relations (see Section 5.3.3). For the sake of completeness, since our approach is a multi-objective one, we also describe the multi-objective local search algorithm employed, namely the Pareto Late Acceptance Hill Climbing (PLAHC) (see Section 5.3.4). While this algorithm is a general-purpose metaheuristic [13], it has been specifically devised for the multi-objective optimization problem at hand.

#### Search Space and Initial Solutions Generation

The search space is represented by a vector for each ambulance type and shift containing the assigned location for the given vehicle. The vectors are ordered, so to break symmetries.

The initial solutions are generated at random. However, one may provide the algorithm with a set of known solutions as well (e.g., the current locations).

#### Cost Function

As outlined in Section 4, our approach adopts a multi-objective methodology to address aspects related to both fairness and efficiency. The cost function components are the fair coverage indicator (Section 4.1), the population and surface coverage (Section 4.2), and the second ambulance distance (Section 4.3). The efficiency KPIs (namely the response time, see Section 4.4) will be accounted for as a post-processing phase by running the simulator on the solutions found by the optimizer.

The observation on the current situation of the vehicles and of the personnel (see Sections 3.2 and 3.3) shows that the number of resources available during the day is significantly greater than the number of resources available during the night. Therefore, it is reasonable to consider the KPIs distinctively for the two situations. Namely, the cost function components are partitioned in day and night indicators as follows: the fair coverage indicators are calculated overall, whereas the population coverage, the surface coverage, and the second ambulance distance are calculated and presented distinclty for the two shifts.

#### Neighborhood Relations

Regarding the neighborhood operator, we consider the possibility of changing the position of one vehicle. More formally, we define the following move:

- *ChangeAmbulance(CA)*: given an ambulance *i*, change its location to *l*. Thus, the move *CA*(*i*, *l*) assigns to the ambulance *i* the new location *l*. Precondition: *l* is different from its previous location.

#### Metaheuristic Algorithm

As already mentioned, the search is driven using PLAHC [13], a multi-objective local search optimization algorithm based on the Late Acceptance Hill Climbing (LAHC) [10].

Algorithm 1 presents the PLAHC procedure. The parameters for this technique are the history length $L_h$ (like the original LAHC), and the maximum number of iterations $i_{max}$. Solutions are stored in a FIFO queue, which is managed as a circular array of size $L_{h}$. The queue is initialized with $L_{h}$ random solutions to ensure diversity. To generate a new candidate solution, a move is applied (line 7) to a solution extracted from the queue (lines 4 and 16). The candidate solution is firstly compared to the reference solution (line 8) and secondly to the next solution in the history (line 11), which allows for a second chance for the candidate solution. If the candidate solution is not accepted, it is discarded (line 14). The comparison of two solutions is done using the Pareto non-dominance relation, which is based on a multi-objective cost function $F = (F_1, F_2, \ldots , F_n)$, where $F(s_1) \prec _P F(s_2)$ if $\exists j \in {1, \ldots , n} : F_{j}(s_1) < F_{j}(s_2) \wedge F_i(s_1) \le F_i(s_2) \forall i \ne j$.

To enhance the exploration of the search space, a second chance is given to the candidate solution by comparing it with a possibly differently structured non-recent solution, similar to the LAHC late acceptance criterion. In this case, the history index is incremented to bypass an immediate check of the candidate solution, which is intended to preserve diversity among solutions (line 13).

The stopping condition for the algorithm considers a maximum iteration limit of 1 million and a tolerance for idle iterations below 20% of the current iteration count, similar to the original LAHC algorithm. Additionally, a timeout feature might be included as an ultimate stopping criterion. When the search is terminated, the history is scanned to provide only the set of non-dominated solutions.

**Algorithm 1:**
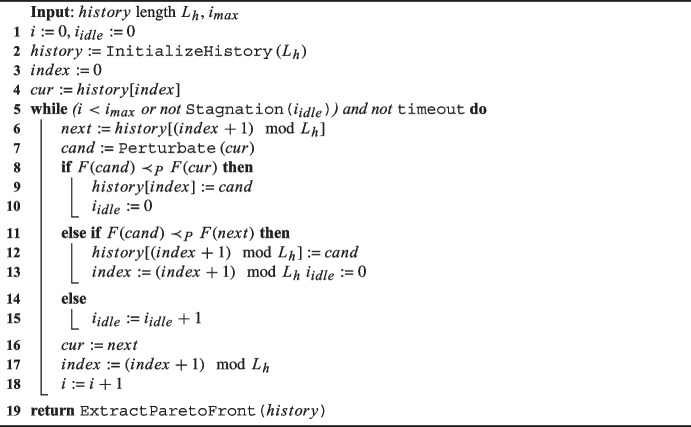
Pseudocode of the PLAHC.

#### Limitations and Assumptions

The optimizer operates on a set of underlying assumptions. The MHs are excluded for the reasons previously detailed in Section 5.2.3. Additionally, the optimizer relies on the relationship between population and the number of cases, thus it is recommended that the results of the optimizer be validated through simulation to obtain a comprehensive understanding of the system. During the optimization process, a small perturbation is utilized as even a minor change to a single ambulance can have a significant impact on the system.

Moreover, each station is assumed to have an infinite capacity, therefore more than one ambulance can be located at a given place. This assumption holds for stations at hospitals. However, for stations located in rural or mountain zones, the arrangement of multiple ambulances at a single station is practically discouraged because of the KPIs related to coverage and equity.

Because of organization requirements, the ambulance shifts are not modified by the optimizer, but only the location of the vehicles as described above.

## Validation of the system

In this section, we report a validation of the system to guarantee that the entire system adheres to real-world scenarios and use cases.

One of the key objectives is to verify that the simulator accurately reflects real-world procedures. This is important to ensure that the simulation results are reliable and can be used to make informed decisions. Additionally, the simulation results should be obtained in a reasonable amount of time. Another important objective is to ensure that the optimizer can provide different high-quality solutions.

In addition to technical objectives, the validation phase also considers the usefulness of the developed tools for the decision-maker, which has been evaluated during a presentation of the system to the EMS executives and practitioners.

In the following, we first illustrate the setup of the experiments (see Section 6.1) and then we present the results of the analysis of the simulator (see Section 6.2) and of the optimizer (see Section 6.3). Finally, we briefly report on the presentation of the system to the EMS stakeholders (see Section 6.4).

### Experimental Setup

To assess the system’s performance, we employ the data concerning emergency calls received from January 2018 to December 2021 (see Section 3.5 for more details on the dataset). Because of privacy concerns and the presence of a confidentiality agreement, the actual dataset from the real world cannot be shared. However, to ensure future comparability and reproducibility of the research, a collection of anonymized instances can be accessed at https://github.com/iolab-uniud/easynet.

Throughout all the tests conducted, the scenario maintains the same number of resources without any changes in comparison to the current situation. Specifically, the number of ambulances and rosters are the same as reported in Table 3. As already mentioned, this is not limitative and scenarios with different resources can be evaluated by changing the input files through the dashboard. Nevertheless, our purpose here is to perform a comparison of the current resource allocation situation.

As for the simulator analysis, the location of the ambulances is also kept unchanged whereas for the optimizer we assess the case of a user that employs the DSS to automatically relocate the ambulances (i.e., the same number of vehicles and the shift configuration). MCs are not considered in the optimization setup due to the requirement for a doctor to operate them. Therefore, we assume that their location is already fixed (usually at one of the hospitals or points of primary care). However, their usage is considered in the simulations.

The other data are as follows: we consider the 215 FVG municipalities and the census subdivision [30] as the zones. The ambulance candidate locations correspond to the town halls of the municipalities. We consider two shifts: a day and a night one. All-day shifts are accounted twice, both as a day and a night shift. The data is prepared by a static preprocessing phase, therefore the system can be easily customized to other regions.

The C++ code of the simulator and the optimizer are compiled with Clang 14 at the O3 optimization level. The computational experiments are conducted on an Apple MacBook Pro 14" 2021 with an Apple M1 Pro processor and 32GB of RAM. Each experiment is run on a single core.

### Simulator Analysis

**Fig. 13:**
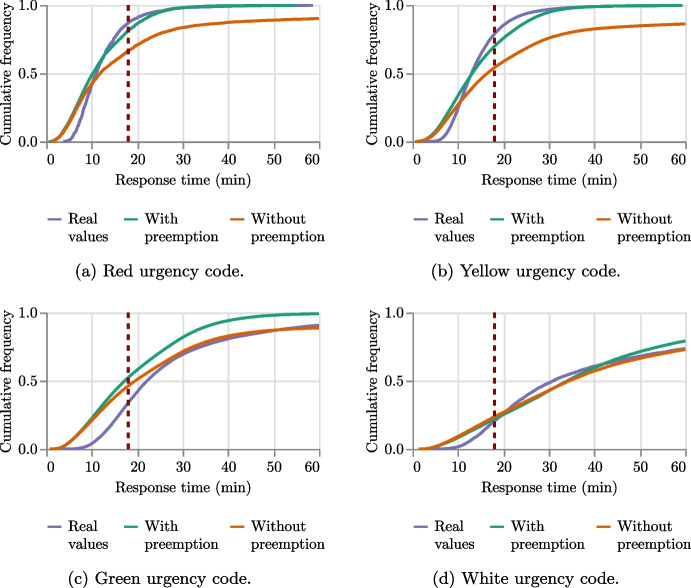
Comparison of the frequency of the response times (in minutes) between real-world response times and simulated ones considering the four urgency codes. Simulations are run in two dispatching modes (i.e., with or without preemption). The 18 minutes threshold is highlighted

The first purpose of this analysis is to check to what extent the simulator adheres to reality. Thus, we compare the simulated times for serving the emergencies of the whole dataset with their real response times. Both the standard dispatching and the non-urgent preemption dispatching strategies are considered. Figure 13 reports, for each urgency code, the curves of cumulative frequency of the response times. These curves show on the *y*-axis the ratio of total emergencies that are served within the number of minutes indicated in the *x*-axis. In the plots, the *x* value corresponding to the 18 minutes threshold is highlighted by a vertical line.

The outcomes reveal a significant alignment between the curve of real values and the simulation executed with the non-urgent preemption dispatching method, particularly for urgency codes red, yellow, and white. While there is a minor deviation from real values in the case of green urgency codes, it is insignificant for our purposes as the decision-maker’s focus is on the realistic processing of higher-priority emergencies.

The second experiment aims at measuring the computational times needed for running the simulation. Indeed, it is very important that the simulation will run as quickly as possible to provide a fast response to the decision-maker.

We run the simulator on a set of random instances of different sizes (i.e., from 7 to 365 days) considering the two dispatching policies and repeating each test five times with different random seeds. The total number of experiments is 5 [runs] $\times$ 11 [temporal horizons] $\times$ 2 [dispatching policies] = 110 tests.

**Fig. 14:**
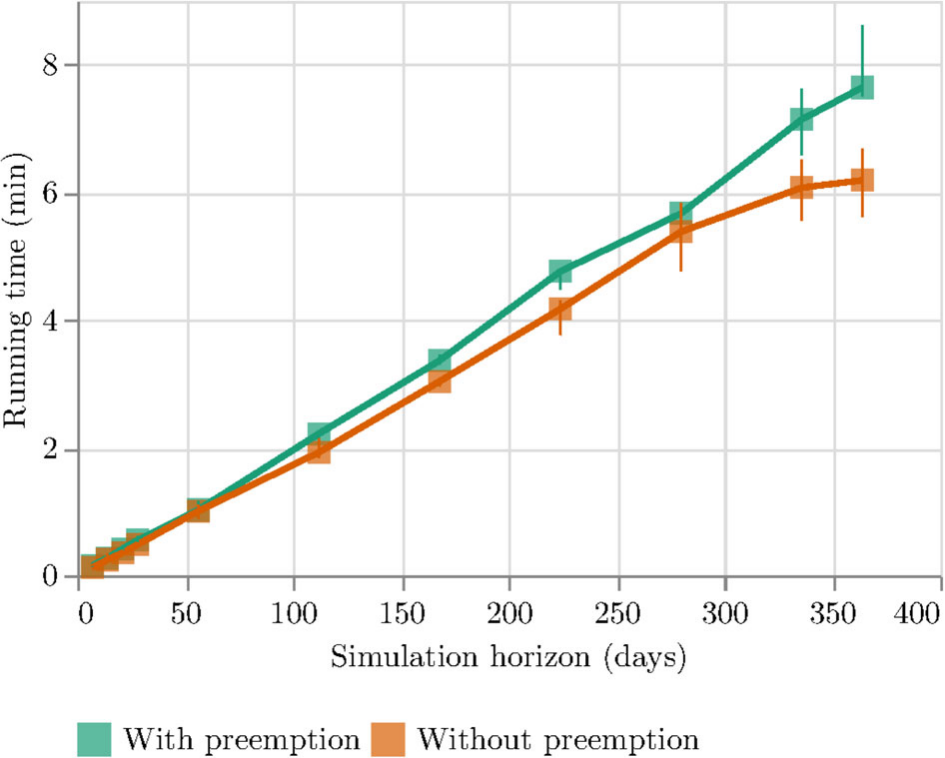
Comparison of the running times (in minutes) of the simulations considering different time horizons (in days) and differentiating between two dispatching modes (i.e., with or without preemption.)

The running times of the simulations are shown in Fig. 14 which reports the median and the range of variation of the simulation running times (*y*-axis) for different time horizons (*x*-axis).

The evidence shows that the times scale almost linearly and that one year of emergencies can be simulated in less than 10 minutes. As might be expected, the management of the non-urgent emergency preemption will require more time than the simpler direct dispatching policy, but the overhead is not that high.

Summarizing the results of this experiment, the simulator provides decision-makers with the opportunity to rapidly and accurately evaluate diverse alternative scenarios under varying stress conditions within a quick simulation timeframe. This capability enables efficient testing and analysis of different strategies in a longer-term simulation horizon.

### Optimizer

In this analysis, similarly to the previous setting, we use the optimizer for determining new ambulance locations on the basis of the current resources.

We run the PLAHC algorithm starting from a pool of random initial solutions. The history length is set at 20 solutions and we fix the maximum number of iterations at 1,000,000.

The results of the algorithm report a Pareto front that comprises 19 different configurations. Each of them improves all the KPIs with respect to the current ambulance location, leading to the conclusion that a better displacement of the ambulance is possible.

**Fig. 15:**
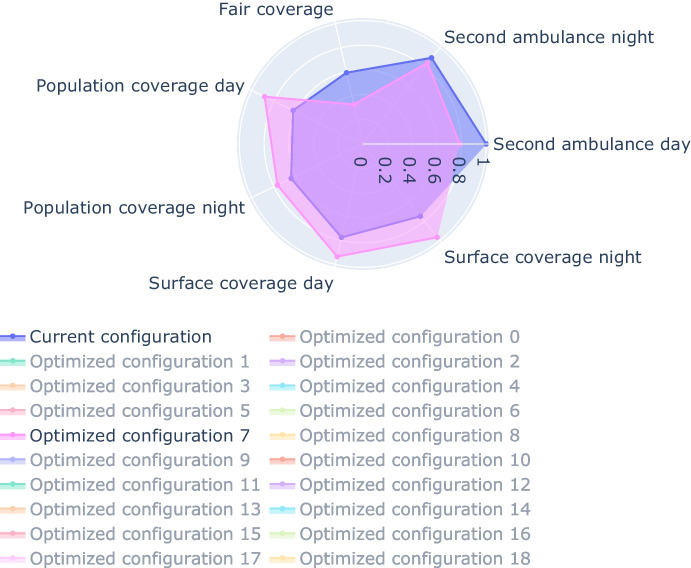
Example of results retrieved from the optimizer. The comparison is among the current configuration and one improved configuration generated from the optimizer

Figure 15 reports an example of results retrieved from the optimizer as shown in the dashboard. In particular, the radar chart shows the cost function components (on a normalized scale). From the dashboard, it is possible to highlight the results of some selected configurations just by clicking on them (e.g., in this case the user selected configuration n. 7).

For instance, the example reported in the figure shows that the highlighted solution enhances all the components compared to the current *as is* state^6^. The population and surface coverage expands in this solution both for the day and the night shift. The fair coverage also improves, as indicated by a value of the Gini index closer to 0. Observing the second ambulance indicator, the KPI is highly improved for the day shift and slightly improved for the night shift.

This tool supports the decision maker to quickly compute different solutions and compare them in a meaningful way. These solutions can then be utilized as scenarios for subsequent simulation analysis, allowing for validation and further evaluation.

The dashboard supports the seamless integration of the optimization and the simulation processes so that also the response times are provided to the decision maker (see Fig. 11b for an example).

### Validation with the Stakeholders

At the end of the development of the DSS, a crucial step involved presenting the tool and the validated results presented in the previous sections to key stakeholders. The individuals involved in this presentation included the Chief Medical Officer of the FVG Regional Healthcare Agency, the Director of the EMS system, and the Manager of the Central Dispatching Center of the EMS.

The stakeholders were provided with a comprehensive overview of the DSS, highlighting its functionalities and the successful validation process, as discussed earlier in this paper. The presentation aimed to demonstrate the DSS’s capabilities in supporting strategic and tactical decision-making processes.

The stakeholders expressed their appreciation for the work undertaken and eagerly anticipated the deployment of the DSS. They recognized the significance of having a robust decision-making tool to aid in the planning and management of emergencies and urgencies. With the need to formulate a new regional plan for emergency and urgency management in the near future, the stakeholders acknowledged the DSS as a valuable asset that would assist them in making informed decisions at both strategic and tactical levels.

This positive reception from the stakeholders further emphasized the importance and the practical relevance of the DSS, paving the way for supporting critical decision-making processes in the EMS domain.

## Conclusion

This study proposes a DSS, composed of a set of software tools, providing insights for Audit and Feedback activities in the EMS context. Differently from existing work, we focus on a large and heterogeneous region featuring various challenges related to its landscape and demographic characteristics. These features led to an analysis of the historical data for driving the design and development of the DSS. In particular, the main challenges were related to providing a fair and efficient service to the whole FVG population under the constraints of limited resources.

Through simulations and optimizations using historical data, the DSS has undergone validation, demonstrating its accuracy, speed, and effectiveness in generating and assessing new allocation scenarios. The feedback received from stakeholders regarding this aspect has been highly positive and encouraging.

One of the key strengths of the DSS presented in this paper is the adaptability of the developed model and simulator. The developed system can be modified to evaluate the impact of a range of scenarios, allowing decision-makers to explore and fine-tune their strategies accordingly. This flexibility enables EMS professionals to better understand the trade-offs between different options, ultimately helping them to make more informed and effective decisions in the management of EMS resources.

The assumptions and the limitations of each component of the DSS are discussed in the specific sections. As a general consideration, the DSS presented in this paper is developed within the Italian context. Consequently, some aspects of the system are specific to this framework, and its application to other case studies may require adaptation to local regulations. Nevertheless, the underlying methodologies and tools can still provide a solid foundation for the development of DSSs tailored to different needs with a little adaptation effort.

Potential extensions of this work include the integration of modules for providing more support for decisions at the operational level (e.g., implementation of different dispatching policies and dynamic relocation). Moreover, the addition of new optimization algorithms should be considered, and the optimization model can be enriched, allowing for the modification of resources (e.g., changing the shifts and dynamically changing the number of available ambulances). Finally, further KPIs as identified by the decision makers can be added to the system either for the simulation and/or for the optimization component.

## Appendix A Definition of the KPIs

### A.1 Fair Coverage

We are given a fleet of ambulances located at positions *J* and operating on *M* municipalities. Each municipality $m \in M$ is further subdivided into $K_m$ census zones. Each census zone $i \in K_m$ has a surface $a_i$ and a population density $d_i$. We denote with *K* the union of all zones, i.e., $K = \bigcup _{m \in M} K_m$.

Each candidate ambulance location $j \in J$ has an isochrone $\textsf {iso}_j$ at *w* minutes (i.e., $w=$18 minutes as for the aforementioned threshold).

If an ambulance is placed at location $j \in J$, then zone $i \in K$ is served by the ambulance at *j* considering the intersection of $a_i$ and $\textsf {iso}_j$. The population of zone *i* covered by the ambulance at *j* is estimated as:

A1 $$\begin{aligned} \textsf {CoveredPopulation}_{ij} = |\textsf {iso}_j \cap a_i| \cdot d_i. \end{aligned}$$

The amount of population that can be covered by an ambulance at location $j \in J$ is denoted by $A_j$ and defined as follows:

A2 $$\begin{aligned} A_j = \sum _{i \in K} \textsf {CoveredPopulation}_{ij} \end{aligned}$$

Additionally, the share of population of a zone $i \in K$ covered by an ambulance located at $j \in J$ can be defined as follows:

A3 $$\begin{aligned} \textsf {CoverageShare}_{ij} = \frac{\textsf {CoveredPopulation}_{ij}}{A_j} \end{aligned}$$

Considering the total population of the region ($\textsf {TotalPopulation} = \sum _{m \in M} p_m$), in the ideal situation each ambulance should serve the same amount of population $\Omega$:

A4 $$\begin{aligned} \Omega = \frac{\textsf {TotalPopulation}}{|J|}. \end{aligned}$$

This means that, in the ideal case, for a given municipality $m \in M$, an expected number of ambulances $\gamma _m = \lceil {\frac{p_m}{\Omega }}\rceil$ would be available.

The *coverage* $X_m$ of a municipality $m \in M$ is measured as the share of service provided to the population of its zones divided by the ideal share of ambulances that would be expected according to its population, that is:

A5 $$\begin{aligned} X_m = \frac{\sum _{i \in K_m} \sum _{j \in L} \textsf {CoverageShare}_{ij}}{\gamma _m \cdot p_m}, \end{aligned}$$

In order to aggregate these values and measure the equality in their distribution, the Gini Index assesses the variation of $X_m$ across different municipalities and is computed as follows:

A6 $$\begin{aligned} G = \frac{\sum _{m_1 \in M} \sum _{m_2 \in M} \left| X_{m_1} - X_{m_2} \right| }{2 \cdot n^2\ \overline{X}} \end{aligned}$$

### A.2 Population Coverage and Surface Coverage

We partition the locations of the fleet of ambulances, into the two sets of $J_{A}$ of ALS ambulances and $J_{B}$ of BLS ones, that is:

A7 $$\begin{aligned} J = J_{A} \cup J_{B} \end{aligned}$$

The population coverage of an ambulance located at $j \in J$ equals the previously defined $A_j$. The final population coverage indicator accounts distinctly for the services of ALS and BLS ambulances as follows:

A8 $$\begin{aligned} \textsf {PopulationCoverage} = \sum _{j \in J_{A}} A_j + 0.7 \cdot \sum _{j \in J_{B}} A_j \end{aligned}$$

On the other hand, the surface coverage for an ambulance located in $j \in J$ is computed as follows:

A9 $$\begin{aligned} S_j = \sum _{i \in K} |\textsf {iso}_j \cap a_i| \end{aligned}$$

As for the overall population coverage indicator, the overall surface coverage indicator accounts distinctly for the two types of ambulances:

A10 $$\begin{aligned} \textsf {SurfaceCoverage} = \sum _{j \in J_{A}} S_j + 0.7 \cdot \sum _{j \in J_{B}} S_j \end{aligned}$$

### A.3 Second Ambulance Distance

As for the second ambulance distance, for each zone $i \in K$ and an ambulance location $j \in J$, the road distance $\delta _{ij}$ between the geographic center of *i* and *j* is computed.

For each zone $i \in K$, we define the set $\Delta _i = \{ \delta _{ij} : j \in J \}$. The values in $\Delta _i = \{ d_{i1}, d_{i2}, \ldots , d_{i|J|} \}$ are sorted in increasing order and $d_{i2}$ is selected as second ambulance for zone *i*.

The KPI refers to the maximum of these values, that is:

A11 $$\begin{aligned} \textsf {SecondAmbulance} = \max _{i \in K} d_{i2} \end{aligned}$$

### A.4 Efficiency Measures

Finally, the response time is measured as the time difference between the occurrence of the emergency call and the arrival at the target.

A12 $$\begin{aligned} \textsf {ResponseTime}_e = \textsf {ArrivalAtTargetTime}_e - \textsf {CallTime}_{e} \end{aligned}$$

The cumulative distribution of the response times for different urgency codes is shown to the user.

## Footnotes

1 An isochrone is the area reachable from an origin location within a given time limit.

2 The dataset consists of data related to the single municipalities, the authors aggregated the data according to the notion of zone (Cities, Rural, etc.) and computed the percentages and the density.

3 The 18 minutes threshold is used as a reference time for the response to time-dependent pathologies such as stroke and heart attack. This value is set as a threshold in the national guidelines issued by the Italian Ministry of Health [49, see indicator D09Z, p. 18].

4 The data on emergency calls is recorded in real-time during the service; however, some fields, particularly the pathology, can be revised after hospitalization.

5 https://github.com/fschuetz04/simcpp20

6 We notice that the population and surface coverage components have to be maximized, whereas the remaining ones have to be minimized.
